# Are Intensive Cares Worthwhile for Breast Cancer Patients: The Experience of an Oncological ICU

**DOI:** 10.3389/fmed.2016.00050

**Published:** 2016-10-31

**Authors:** Virginie Destrebecq, Ameye Lieveke, Thierry Berghmans, Marianne Paesmans, Jean-Paul Sculier, Anne-Pascale Meert

**Affiliations:** ^1^Service des soins intensifs et urgences oncologiques & oncologie thoracique, Institut Jules Bordet, Université Libre de Bruxelles (ULB), Brussels, Belgium; ^2^Data Centre, Institut Jules Bordet, Université Libre de Bruxelles (ULB), Brussels, Belgium

**Keywords:** breast cancer, intensive care, survival

## Abstract

**Purpose:**

One among seven women will present with breast cancer for which major therapeutic advances led to a significant increase in survival and cure rates. During or after cancer treatment, severe complications may occur requiring admission in intensive care unit (ICU). Intensivists could be reluctant for accepting cancer patients in the ICU, and there are very few data about causes of admission and prognosis of patients with breast cancer admitted in the ICU for an acute complication. Our study seeks to determine, in a population of patients with breast cancer, the main causes for ICU admission and the predictors of death during hospital stay and prognostic factors for survival after hospital discharge.

**Methods:**

This retrospective study includes all unplanned ICU admissions of patients with breast cancer in a cancer hospital from January 1, 2009 to December 31, 2014. To search for predictive factors of death during hospitalization, Mann–Whitney or Fisher Exact (or chi-square) tests were used for continuous variables or categorical variables, respectively. A logistic regression model was applied for multivariate analysis. Multivariate analysis of prognostic factors for survival after hospital discharge was performed with a Cox’s proportional hazards model.

**Results:**

Of 1586 ICU admissions during the study period, 282 (18%) concerned breast cancer of which 175 met the inclusion criteria. The main causes of admission were of cardiovascular (26%), respiratory (19%), neurologic (19%), or infectious (14%) origin. ICU death rate was 15% and, overall, 28% of the patients died during hospitalization. The median survival time after hospitalization was 12.8 months (95% CI: 8.2–20.7). Independent predictors of death during hospitalization were the sequential organ failure assessment (SOFA) score (OR 1.36, 95% CI 1.15–1.60), high GPT values (OR 3.70, 95% CI: 1.52–9.03), and cardiovascular disease (OR 0.23, 95% CI: 0.06–0.86). Independent predictors of death after hospital discharge were metastatic disease (HR 7.90, 95% CI 3.69–16.92), high GOT value (HR 3.22 95% CI: 1.93–5.36), simplified acute physiology score (SAPS) (HR 1.95 95% CI: 1.21–3.16), and therapeutic limitations during the first 24 h after ICU admission (HR 8.52 95% CI: 3.66–19.87).

**Conclusion:**

Independent predictors of death during hospitalization were related to the acute complications (SOFA score, GPT level and cardiovascular-related admission) while cancer parameters retained their prognostic significance for survival after hospital discharge (metastatic disease, therapeutic limitations).

## Introduction

In Europe, approximately one in seven patients admitted to an intensive care unit (ICU) are presenting with a cancer, mainly solid tumors (1). Reasons for admitting cancer patients in the ICU are multiple, including complications due to cancer or its treatment as well as other diseases unrelated to the neoplastic disease. The majority of publications on cancer patients in ICU are concerning populations with mixed types of cancers patients with hematological malignancies or lung cancer (2, 3). Survival of cancer patients admitted to the ICU is influenced by the physiological disturbances caused by the complications leading to ICU admission. Cancer characteristics recovered all their importance for further prognosis only after hospital discharge. In this setting, a discussion between the intensivist and the treating oncologist is of particular importance. Both have to integrate the therapeutic option, and the possibility of cancer control as well as the prognosis linked to the acute complication before admitting in the ICU and/or determining the potential limits of the critical management.

Breast cancer is the leading cause of cancer in women with an incidence of 110/100,000 in Belgium in 2013 (4). Meaningful improvement in survival and cure rates are been obtained with newer systemic therapies. Even for metastatic breast cancer, which is unlikely to be cured, median overall survival is reaching 2 years, with a range from a few months to many years. These women can thus have complications related or not to the tumors and its anticancer treatment (febrile neutropenia and septic shock, cardiac failure, respiratory failure, new adverse events from innovative biological therapies…) that worsens the prognosis but can be improved with intensive care support.

There are few data published about causes of admission and prognosis of patients with breast cancer admitted in the ICU for an acute complication. Having a better knowledge of the diseases leading to ICU admission and of the prognosis of such patients might help in triage decision as well for intensivists and oncologists. A retrospective study, which aimed to validate the prognostic value of the APACHE II score in 66 patients with breast cancer identified respiratory failure as the primary reason for ICU admission (29%), pericardial effusion (23%), and cardiac arrhythmias (12%) being in second and third positions (5). Prognostic factors for hospital mortality were the APACHE II score, presence and number of metastases, reason of ICU admission and length of ICU stay. Another small study, including patients with gynecological (ovarian, endometrial, and cervical) and 11 breast cancer, identified sepsis (95%), and respiratory failure (37%) as the main reasons for ICU admission. The ICU mortality rate was 31%, overall mortality of the patients during hospitalization was 58%, and 6-month mortality rate was 68% (6).

The aim of the present study was to determine the main causes of ICU admission in patients with breast cancer treated in an academic cancer center. We also sought to identify predictors of death during hospitalization and prognostic factors for survival after hospital discharge in order to help further selection of breast cancer patients, which may benefit from ICU admission.

## Patients and Methods

This retrospective study is based on the medical records of patients consequently admitted to the ICU of an academic cancer center, between January 1, 2009 and December 31, 2014. In our oncological center, ICU admission occurred after extensive discussion between the intensivist and the oncologist who take care of the patient, based on the oncological therapeutic plan, the ICU indications and the patient’s wish. Inclusion criteria were all breast cancer patients admitted in the ICU for an acute, potentially life-threatening, complication (respiratory failure, shock, arrhythmias…). Patients with planned admissions were excluded. Planned admissions are defined as monitoring of a treatment at risk (allergic reaction, arrhythmias…) or postoperative monitoring of planned surgery. We also excluded patients with another active cancer. This study was approved by the local ethical committee of the Institute Jules Bordet on October 16, 2014.

For each admission, the following data were retrospectively retrieved from the computerized charts:

– Age, sex, comorbidities according to the Charlson’s score (giving one point each: myocardial infarct, congestive heart failure, peripheral vascular disease, dementia, cerebrovascular disease, chronic lung disease, connective tissue disease, ulcer, chronic liver disease, and diabetes; two points each: hemiplegia, moderate or severe kidney disease, diabetes with end organ damage, tumor, leukemia, and lymphoma; three points each: moderate or severe liver disease; six points each: malignant tumor, metastasis, and AIDS).
– Date of first cancer diagnosis, cancer stage (metastatic or not).
– Anticancer treatment (with all systemic anticancer therapy – chemotherapy or targeted therapy in the 30 days before admission to the ICU).
– Cancer phase according to the Australian classification (7): diagnostic phase when the patient is under work-up for newly diagnosed neoplasia; curative phase when a curative treatment is started or when the patient is cured or in complete remission; control phase when the therapeutic project can lead to a temporary remission with improved survival but without possibility of cure; pivotal phase when a disease-oriented treatment is no longer available requiring a switch to palliative care; palliative phase when only comfort care can be proposed.
– Cancer status has been divided in five categories: induction therapy, complete remission (defined as complete disappearance of all manifestations of disease found on physical examination and radiologic study) under treatment or without active therapy, partial remission (defined as 50% or greater reduction in the measurable parameters of tumor growth as may be found on physical examination and radiologic study), stable disease, or progressive disease according to the last disease work-up.
– Date and reason for ICU admission; when multiple reasons of ICU admission being found, only the initial event responsible for the physiological disturbances was considered.
– Biological data: transaminases (GOT, GPT), alkaline phosphatases (PAL), lactate dehydrogenase (LDH), C-reactive protein (CRP), lactate, hemoglobin levels, neutrophils, and lymphocyte count.
– Treatment limitations distinguishing between preexisting limitations, limitations occurring during the first 24 h after ICU admission and limitations occurring after 24 h in the ICU. We determined the reasons for these limitations: neoplasia, multifactorial (including a neoplastic cause, treatment complication, and/or infectious complication), and other reason.
– Life supporting techniques used in the ICU: invasive and/or non-invasive ventilation, vasoactive amines, dialysis, and cardiopulmonary resuscitation.
– Length of ICU and hospital stay, date, and cause of death (for the surviving patients, the date of last follow-up was used).

### Statistical Analyses

We assessed two outcomes: (1) hospital death (binary outcome), i.e., alive at the end of hospitalization vs. died during the hospitalization (in the ICU or in another hospital unit) and (2) time to death after hospital discharge in the patients alive at the end of hospitalization (time to event outcome). In patients with more than one ICU admission, we considered only the first admission.

To assess differences between patients who died during hospitalization and those alive at end of hospitalization, the Mann–Whitney test was used for continuous variables and the Fisher Exact or chi-square test for categorical variables. Multivariate analysis for predictors of in-hospital death was done with a logistic regression model.

To assess prognostic factors for survival after hospital discharge, we calculated hazard ratios (HRs) using Cox’s proportional hazards model. Continuous variables were dichotomized using the median in all ICU admitted patients.

For both the multivariate model of hospital mortality (first outcome variable) as the multivariate model for survival after hospital discharge (second outcome variable), we considered only variables with a univariate *p*-value <0.20 as possible predictor for the respective outcome, and performed stepwise variable selection.

All statistical analyses were performed using SAS 9.3. All reported *p-*values are two-tailed and a *p-*value <0.05 was considered as statistically significant.

## Results

Between January 1, 2009 and December 31, 2014, there were 1586 ICU admissions of whom 282 (18%) concerned patients with breast cancer. Of those 282 patients, 107 were ineligible for the study: therapeutic monitoring (*n* = 46), planned postoperative monitoring (*n* = 36), more than one active cancer at the time of admission (*n* = 19), and other causes (*n* = 6). Due to multiple admissions in the same patient, 159 patients accounted for the 175 admissions. All admissions were considered for the description of causes of ICU admission but only the first stay in the ICU was considered for hospital mortality and survival after discharge analyses.

Table 1 shows the main patients’ characteristics. Table 2 shows the principal reason for ICU admission. They were in decreasing frequencies from cardiovascular (26%), respiratory (19%), neurologic (19%), metabolic (10%), infectious (8%), renal (8%), digestive (5%), and hemodynamic (5%) origins. Table 3 summarizes the therapeutic limitations decided for 44 admissions. These limitations were mostly due to the cancer evolution found after ICU admission or during ICU stay.

**Table 1:** **Characteristics of breast cancer patients admitted in the ICU**.

Patient’s characteristics	*N*	%
Women^a^	159	100
Median age^a^ (min–max)	57 (27–91)	
Metastatic stage^b^	134	77
Cancer status^b^		
• Induction treatment	15	9
• Complete remission	26	15
• Partial remission	1	<1
• Stable	31	18
• Progression	102	58
Cancer phase^b^		
• Diagnostic	4	2
• Curative	42	24
• Control	109	62
• Pivotal	19	11
• Palliative	1	<1
Anticancer treatment ongoing 30 days before ICU^b^	75	43
• Chemotherapy	67	38
• Targeted therapy	7	4
• Missing data	1	<1
Median Glasgow score^b^ (min–max)	15 (5–15)	
Median Charlson score^b^ (min–max)	8 (3–15)	
Median SAPS 2 score^b^ (min–max)	34 (13–81)	
Median SOFA score^b^ (min–max)	2 (0–11)	
Neutropenia^b^	14	8

*^a^N = 159 patients*.

*^b^N = 175 admissions*.

**Table 2:** **Causes of admission in the ICU of breast cancer patients**.

Causes of admission	*N*	%
Cardiovascular	45	26
• Cardiac failure	12	7
• Thrombosis and pulmonary embolism	10	6
• Syncope	8	5
• Arrhythmia	8	5
• Pericarditis	4	2
• Myocardial ischemia	1	<1
• Cardiac arrest	1	<1
• Hypertensive emergency	1	<1
Respiratory	34	19
• Pneumonia	10	6
• Severe respiratory distress of multifactorial causes	9	5
• Pleural effusion	8	5
• Chronic obstructive pulmonary disease exacerbation	3	2
• Pneumothorax	2	1
• Severe asthma	1	<1
• Hemoptysis	1	<1
Neurological	33	19
• Seizures	14	8
• Coma/impaired alertness	5	3
• Intracranial hypertension	5	3
• Cerebral hemorrhage	4	2
• Stroke	3	2
• Paralytic syndrome	2	1
Metabolic	17	10
• Electrolytic disorders	13	8
• Metabolic acidosis, ketoacidosis coma, hypoglycemia, and decompensated diabetes	4	2
Renal disease	14	8
• Acute renal failure	14	8
Infectious disease	14	8
• Sepsis	12	7
• Erysipelas	1	<1
• Fever of unknown origin	1	<1
Digestive	9	5
• Hepatic failure	4	2
• Hemorrhage	3	2
• Abdominal compartment syndrome	2	1
Hemodynamics	9	5
• Septic shock	6	3
• Anaphylaxis	2	1
• Hemorrhagic shock	1	<1

**Table 3:** **Life support techniques limitations for breast cancer patients admitted in the ICU**.

Life support techniques, limitations, and causes	*N*: 175	%
Limitations	44	25
• Limitations at ICU admission or during the first 24 h	18	10
• Limitations beyond 24 h	26	15
Causes of limitations		
• Neoplasia	34	19
• Multifactorial	5	3
• Others	4	2
• Unknown by missing data in the chart	1	<1

Life supporting techniques were used during 105 admissions: 56 non-invasive ventilations, 20 vasoactive amines, 16 invasive ventilations, 10 continuous venovenous hemodiafiltration (CVVHDF), and 3 cardiopulmonary resuscitations.

Forty-four (28%) patients died during the hospital stay mainly for reasons related to neoplasia (17%). Twenty-four (15%) patients died in the ICU, and 20 patients after ICU discharge during the hospital stay. Table 4 shows the results of the univariate analyses for hospital mortality. The following variables were significantly associated with hospital mortality in univariate analysis: metastatic disease, cancer phase at ICU admission, cancer status, causes of admissions, FiO2, SAPS II and SOFA scores, lactate, GOT, GPT, and CRP levels; in addition to those, we added in the multivariate model, the variables with a *p-*value <0.2: SpO2, PAL hemoglobin level, lymphocytes count, and ICU limitations <24 h.

**Table 4:** **Univariate analysis for hospital mortality of breast cancer patients admitted in the ICU (*N* = 159)**.

	All patients (*N*: 159)	Alive at the end of the hospitalization (*N*: 115)	Died in the ICU or at the end of the hospitalization (*N*: 44)	*p*-value
Age, median ± SD	58.5 ± 13.7	58.1 ± 13.7	59.6 ± 13.5	0.54
Metastatic disease				
No	39 (25%)	35 (30%)	4 (9%)	**0.003**
Yes	120 (75%)	80 (70%)	40 (91%)	
Cancer phase at ICU admission				0.008*
Diagnosis	4 (3%)	1 (<1%)	3 (7%)	0.06
Curative	40 (25%)	35 (30%)	5 (11%)	**0.01**
Control	97 (61%)	69 (60%)	28 (64%)	0.67
Pivot	17 (11%)	9 (8%)	8 (18%)	0.08
Palliative	1 (<1%)	1 (<1%)	–	1
Cancer status				0.048*
Induction	15 (9%)	11 (10%)	4 (9%)	1
Complete remission	25 (16%)	22 (19%)	3 (7%)	0.09
Partial remission	1 (<1%)	1 (<1%)	–	1
Stable	27 (17%)	23 (20%)	4 (9%)	0.16
Progression	91 (57%)	58 (50%)	33 (75%)	**0.007**
Causes of admissions				0.002*
Respiratory	28 (18%)	19 (17%)	9 (20%)	0.56
Cardiovascular	42 (26%)	38 (33%)	4 (9%)	**0.002**
Neurologic	31 (20%)	21 (18%)	10 (23%)	0.51
Digestive	8 (5%)	2 (2%)	6 (14%)	**0.006**
Infectious	13 (8%)	9 (8%)	4 (9%)	0.75
Hemodynamic	9 (6%)	5 (4%)	4 (9%)	0.26
Metabolic	16 (10%)	14 (12%)	2 (5%)	0.24
Renal disease	12 (8%)	7 (6%)	5 (11%)	0.32
Antineoplastic therapy within 30 days before ICU admission				
No	92 (58%)	66 (57%)	26 (59%)	0.85
Yes	67 (42%)	49 (43%)	18 (41%)	
Vital parameters				
Sp O_2_				
*N*	156	112	44	0.18
Median (min–max)	95 (33–100)	95 (33–100)	94 (76–100)	
Fi O_2_				
*N*	156	112	44	**<0.001**
Median (min–Q1–Q3–max)	21 (21–21–21–100)	21 (21–21–21–80)	21 (21–21–30–100)	
Lactate
*N*		54	27	**0.002**
Median (min–max)	8118 (6–139)	15 (6–89)	25 (12–139)	
GOT				
*N*	148	109	39	
Median (IU/l) (min–max)	36 (8–1776)	29 (8–1776)	84 (15–1539)	**<0.001**
GPT				
*N*	148	109	39	
Median (IU/l) (min–max)	24 (5–2130)	21 (5–275)	48 (9–2130)	**<0.001**
PAL				
*N*	147	108	39	
Median (IU/l) (min–max)	231 (35–3319)	213 (36–2442)	272 (35–3319)	0.14
LDH				
*N*	100	76	24	
Median (IU/l) (min–max)	489 (164–29999)	481 (164–29999)	560 (266–23913)	0.23
Hemoglobin				
*N*	157	113	44	
Median (g/dl) (min–max)	11.9 (5.2–18.3)	11.9 (6.5–18.3)	10.9 (5.2–15.1)	0.08
Neutrophils				
*N*	134	101	33	
Median/μl (min–max)	5655 (120–37410)	5590 (540–37410)	6080 (120–23490)	0.31
Lymphocytes				
*N*	129	99	30	
Median/μl (min–max)	960 (100–13800)	1000 (100–13800)	735 (190–3910)	0.17
CRP				
*N*	153	111	42	
Median (mg/l) (min–max)	37.6 (0.2–413)	27.1 (0.3–325.4)	97.5 (0.2–413)	**<0.001**
Charlson’s score				
*N*	156	114	42	
Median (min–max)	8 (3–15)	8 (3–15)	8 (5–14)	0.4
SAPS II score				
*N*	154	111	43	
Median (min–max)	35 (13–81)	33 (13–66)	43 (18–81)	**<0.001**
SOFA score				
*N*	155	111	44	
Median (min–max)	2 (0–11)	1 (0–10)	4 (0–11)	**<0.001**
ICU limitations (<24 h)				
No	140 (89%)	104 (91%)	36 (84%)	0.25
Yes	17 (11%)	10 (9%)	7 (16%)	
Missing data	2			

*In categorical variables with more than two categories, we performed an overall test to assess whether there was an association between the categorical variable (considering all categories) and the outcome: first *p*-value. And then we tested each category vs. all others: *p*-value for each category.

*p < 0.2 are in bold*.

In multivariate analysis, we found three statistically independent predictors of death during hospitalization: SOFA score (OR 1.36 per one-unit increase, 95% CI: 1.15–1.60; *p* < 0.001), and GPT above the median value (OR 3.7, 95% CI: 1.52–9.03, *p* = 0.004) as predictive factors of poor outcome, while cardiovascular disease as cause of the admission (OR 0.23, 95% CI: 0.06–0.86, *p* = 0.03) being a predictive factor of better outcome (Table 5).

**Table 5:** **Multivariate analysis for hospital mortality of breast cancer patients admitted in the ICU**.

	Odd ratio	95%CI	*p*-value
SOFA score	1.36	1.15–1.60	<0.001
GPT > 24 (median)	3.7	1.52–9.03	0.004
Cardiovascular admissions	0.23	0.06–0.86	0.03

During the study period, 115 patients were discharged from the hospital. Median survival after hospital discharge was 12.8 months (95% CI: 8.2–20.7). Table 6 shows the results of the univariate analyses for prognostic factors for survival after hospital discharge. The following variables were significantly associated with survival in univariate analyses: metastatic disease, cancer phase at ICU admission, cancer status, GOT, PAL, hemoglobin and lactate dehydrogenase (LDH) levels, SAPSII and Charlson scores, ICU life support limitations <24 h; in addition to those, we added in the multivariate model the variables with a *p-*value <0.2: Fi O_2_, GPT, and CRP levels, SOFA score, lymphocytes count. In multivariate analysis (Table 7), we found four statistically independent prognostic factors for survival after hospital discharge: metastatic disease (HR 7.9, 95% CI:3.69–16.9, *p* < 0.001), GOT above the median value (HR 3.22 95% CI:1.93–5.36, *p* < 0.001), SAPS score above the median value (HR 1.95 95% CI:1.21–3.16, *p* = 0.006), and ICU life support limitations at or during the first 24 h of ICU admission (HR 8.52 CI 95%:3.66–19.87, *p* < 0.001).

**Table 6:** **Univariate analysis for survival after discharge (*N* = 115)**.

		Hazard ratio (95% CI)	*p*-value
Age	>58 vs. ≤58	1.05 (0.67–1.64)	0.84
Metastatic extension	Yes vs. no	8.31 (3.94–17.51)	**<0.001**
Phase of cancer^a^	Curative vs. other than curative	0.16 (0.08–0.31)	**<0.001**
	Control vs. other than control	3.43 (2.01–5.87)	**<0.001**
	Pivotal vs. other than pivotal	4.11 (2.02–8.35)	**<0.001**
Status of cancer^b^	Induction vs. other than induction	0.21 (0.07–0.67)	**0.009**
	Remission vs. other than remission	0.38 (0.18–0.78)	**0.009**
	Stable vs. other than stable	0.92 (0.54–1.57)	0.77
	Progression vs. other than progression	3.47 (2.16–5.56)	**<0.001**
Causes of admission^c^	Respiratory vs. other than respiratory	1.17 (0.67–2.06)	0.59
	Cardiovascular vs. other than cardiovascular	0.47 (0.28–0.79)	**0.004**
	Neurological vs. other than neurological	1.17 (0.65–2.09)	0.6
	Infectious vs. other than infectious	0.45 (0.16–1.23)	0.12
	Hemodynamic vs. other than hemodynamic	1.35 (0.49–3.71)	0.56
	Metabolic vs. other than metabolic	1.98 (1.09–3.62)	**0.03**
	Renal vs. other than renal	5.87 (2.42–14.23)	**<0.001**
Antineoplastic treatment	Yes vs. no	1.04 (0.66–1.63)	0.87
Sp O_2_%	>95 vs. ≤95	0.95 (0.60–1.50)	0.82
Fl O_2_%	>21 vs. 21	1.52 (0.87–2.66)	0.14
Lactate (mg/dl)	>18 vs. ≤18	0.78 (0.39–1.48)	0.42
GOT IU/l	>36 vs. ≤36	2.40 (1.52–3.81)	**<0.001**
GPT IU/l	>24 vs. ≤24	1.47 (0.93–2.34)	0.1
PAL IU/l	>231 vs. ≤231	1.90 (1.20–3.00)	**0.007**
LDH IU/l	>489 vs. ≤489	1.94 (1.09–3.46)	**0.03**
Hemoglobin (g/dl)	>11.9 vs. ≤11.9	0.49 (0.31–0.77)	**0.002**
Neutrophils/μl	>5655 vs. ≤5655	0.82 (0.51–1.34)	0.43
Lymphocytes/μl	>960 vs. ≤960	0.64 (0.40–1.04)	0.07
CRP (mg/l)	>37.6 vs. ≤37.6	1.50 (0.95–2.36)	0.08
Charlson’s score	>8 vs. ≤8	1.98 (1.26–3.11)	**0.003**
SAPSII score	>35 vs. ≤35	1.66 (1.05–2.62)	**0.03**
SOFA score	>2 vs. ≤2	1.55 (0.97–2.47)	0.07
ICU life support techniques limitation <24 h	Yes vs. no	7.32 (3.38–15.87)	**<0.001**

*^a^Insufficient cases to assess diagnostic and palliative phases*.

*^b^Insufficient number of cases to assess partial remission*.

*^c^Insufficient cases to assess the digestive admissions*.

*p < 0.2 are in bold*.

**Table 7:** **Multivariate Analysis for mortality to hospital discharge of breast cancer patients admitted in the ICU**.

	Hazard ratio	95% CI	*p*-value
Metastatic extension (yes vs. no)	7.90	3.69–16.92	<0.0001
GOT > 36 (vs. ≤36)	3.22	1.93–5.36	<0.0001
SAPS > 35 (vs. ≤35)	1.95	1.21–3.16	0.0062
Life support limitations <24 h (yes vs. no)	8.52	3.66–19.87	<0.0001

## Discussion

Eighteen percent of unplanned ICU admissions for an acute complication in an academic cancer hospital concerned patients with breast cancer, mainly for cardiovascular and respiratory diseases. The mortality rates in the ICU and during the hospital stay were 15 and 28%, and the median survival after discharge from the hospital was 12.8 months. Independent predictors of mortality during hospitalization were related to the acute complications: SOFA score and high GPT value and cardiovascular-related. After discharge from the hospital, independent prognostic factors for survival were more related to the cancer characteristics: metastatic disease, high GOT value, SAPS II score, and ICU life support limitations during the first 24 h after admission.

Breast cancer is the leading cause of cancer in women. One among seven women will present during their life this potentially devastating cancer. New therapies, including aggressive chemotherapy for localized operable cancer and for metastatic diseases, led to a significant improvement in survival and cure rates. Even for metastatic breast cancers that are unlikely to be cured, survival can reach many years. Whatever considering direct cancer complications or acute and delayed adverse events linked to its treatment, women with breast cancer are likely to be admitted during their lifetime in an ICU. We are confirming the importance of this problem in our series with 18% of the ICU admission occurring in patients with breast cancer.

When admitting cancer patients into an ICU, there is a need for discussion between the intensivist, according to the prognosis of the acute complication, and the oncologist taking into account the therapeutic option that could be delivered during or after the ICU stay. However, few data are available on that important topic that can affect both cancer center and general hospitals. To the best of our knowledge, our study is the first large series specifically dealing with breast cancer patients admitted in an ICU.

A systematic review of the literature (8) evaluated the survival of patients with solid tumors admitted to ICU. It identified 25,339 admissions between 1997 and 2011. Eight publications do not specify the types of solid tumors. Publications specifying the type of tumor identified altogether 8225 patients including 221 with breast cancer (3%). The limited number of breast cancer patients at the difference of the present series could be linked to a selection bias as only a minority of ICU was from oncological centers. The only study comparable to our series, dealing only with breast cancer patients, find as main causes of admission: respiratory failure (29%), pericardial effusion, and arrhythmias (6%) (5). Other publications on mixed cancer populations included small samples of patients with breast cancer. No specific data for these breast cancer patients can be derived except the same type of complications leading to ICU admission were observed: respiratory failure and sepsis being the leading cause of admission (1, 2, 6, 9). The high rates of cardiovascular complications in our series may be explained by the cardiotoxic effect of some drugs used to treat breast cancer as anthracyclines derivatives and trastuzumab that are more commonly used today for (neo)adjuvant chemotherapy than in the period considered in the Headley’s series (5). Effectively, five cases of cardiac failure linked to chemotherapy (anthracyclines, taxanes, and ciplatin-5FU), trastuzumab, and radiotherapy were recorded. The taxanes were implicated in the development of two cases of arrhythmia and aromatase inhibitors in three cases of thromboembolism.

In the systematic review previously mentioned (8), ICU mortality for all solid tumors ranged from 4.5 to 85%, hospital mortality from 4.6 to 77%, and 1-year mortality from 36 to 88%. A study combining gynecological and breast cancer patients found an ICU mortality of 31% and 6-month mortality of 68% (6). It is difficult to make a meaningful comparison due to the different case-mixes and the different definition of 1-year mortality or survival after hospital discharge. In our series, we observed a lower mortality rate. One explanation could be a kind of patient selection because in our institution, according to the cancer evolution and the patient’s wishes, decision for ICU life support limitations are taken before any ICU admission. Very few patients were admitted at pivotal or palliative phases, variables, which were not taken into account in the previous publications. Supporting this argument, we found that ICU life support limitations decision taken early in the course of the ICU stay was associated with a poor outcome. Those decisions were mainly taken in front of cancer evolution discovered after ICU admission.

Life support techniques used during the ICU stay, invasive mechanical ventilation, amines support, and cardiopulmonary resuscitation, were associated with a higher mortality rate as in other publications (9, 10). We decided to not include them in the predictive and prognostic factors analyses because we only considered variables available at ICU admission except for gravity scores. In various publications, the prognosis in ICU and hospital stay of cancer patients is mainly influenced by the physiological disturbances caused by the complications leading to ICU admission (11–13). For breast cancer in particular, a relationship between hospital mortality and APACHE II score was reported (5) despite limited sensitivity (54%) for hospital mortality. We confirmed in this study that general ICU gravity scores, reflecting the importance of physiological disturbances, are predicting hospital mortality but are also of prognostic value for survival after hospital discharge. In addition, we identified some easily measurable variables like GPT as predictor of hospital mortality and GOT as prognostic factor for survival after hospital discharge. As the literature is scarce concerning the prognostic value of GOT/GPT in cancer patients admitted into ICU, we postulate that an increase in liver enzymes occurred in the context of multiple organ failure, secondary to the acute complication but can also be, in a limited number of patients, due to liver dysfunction in the context of metastasis in the liver, yet already present beforehand for some people. This relationship had already been observed in a previous study on colorectal cancer from our group (13).

We confirmed the findings from previous publications that cancer characteristics retained their prognostic value for survival after hospital discharge, identifying in the present study metastatic disease as an independent prognostic factor. Indeed, a relationship was reported between the metastatic cancer extent and the hospital mortality (3, 10, 14).

Some limitations of our work have to be reported. The retrospective nature of the study could be associated with a selection bias. Nevertheless, all patients consecutively admitted to the ICU were recorded and all medical charts were available at time of the study, and only a few data were missing.

This a single-center study carried out in a dedicated Belgian cancer center recruiting a large number of patients with breast cancer. This raises the question of the generalizability of our results. It would be interesting to conduct a prospective multicenter study with a larger number of patients and including oncological and general ICUs as well for confirming our data.

## Conclusion

This is, to our knowledge, the first large sample-size study assessing unplanned ICU admission of patients with breast cancer. The ICU mortality was 15%, and the overall mortality of the patients during hospitalization was 28%. The prognostic factors for mortality during hospitalization were related to the acute complications leading to critical care: SOFA score, GPT level, and cardiovascular-related admission. The prognostic factors of mortality after hospitalization were more related to the cancer characteristics: metastatic disease, GOT level, SAPS score, and therapeutic limitations during the first 24 h after admission.

## Author Contributions

A-PM, AL, TB, J-PS, MP, and VD gave substantial contributions to the conception and design of the work, the acquisition, analysis, and interpretation of data for the work. They all revised the manuscript and approved the final version. They agreed to be accountable for all aspects of the work in ensuring that questions related to the accuracy or integrity of any part of the work are appropriately investigated and resolved.

## Conflict of Interest Statement

The authors declare that the research was conducted in the absence of any commercial or financial relationships that could be construed as a potential conflict of interest.
